# Synthesis of 4′,7-Diacetoxyapigenin and Its Apoptotic Induction in Human Hep G2 Cells

**DOI:** 10.3390/ijms11051991

**Published:** 2010-04-30

**Authors:** Keyong Xu, Feng Liu, Benguo Liu, Han Gao, Zhengxiang Ning

**Affiliations:** 1 College of Light Industry and Food Science, South China University of Technology, Guangzhou 510640, China; E-Mail: fezhning@scut.edu.cn (Z.N.); 2 The Key Laboratory of Chemical Biology, Guangdong Province, Graduate School at Shenzhen, Tsinghua University, Guangdong 518055, China; E-Mail: liu.feng@sz.tsinghua.edu.cn (F.L.); 3 School of Food Science, Henan Institute of Science and Technology, Xinxiang 453003, China; E-Mails: zzgclbg@126.com (B.L.); gh@hist.edu.cn (H.G.)

**Keywords:** apoptosis, cell cycle, Hep G2, 4′,7-diacetoxyapigenin

## Abstract

In this study, 4′,7-diacetoxyapigenin [4-(7-acetoxy-5-hydroxy-4-oxo-4H-chromen-2-yl) phenyl acetate] was synthesized for the first time. Its chemical structure was identified by UV, ESI-MS, ^1^H and ^13^C-NMR. It could inhibit the proliferation of Hep G2 cells in a dose-dependent manner and induce the significant increase of the G0/G1 cell population. After treatment by 4′,7-diacetoxyapigenin, phosphatidylserine of Hep G2 cells could significantly translocate to the surface of the membrane. The increase of an early apoptotic population was observed by both annexin-FITC and PI staining. It was concluded that 4′,7-diacetoxyapigenin not only induced cells to enter into apoptosis, but also affected the progress of the cell cycle.

## 1. Introduction

Apoptosis is a highly regulated cell death process with characteristic biochemical features [1,2] and membrane-bond apoptotic bodies [3]. It occurs both during normal development and under certain pathological conditions in metazoans, and plays a crucial role in the maintenance of tissue homeostasis by the selective elimination of excessive cells [4,5]. Evasion of apoptosis is an essential hallmark of cancer [6]. Genetic changes resulting in loss of apoptosis or derangement of apoptosis-signalling pathways in the transformed cells are likely to be critical components of carcinogenesis [7,8]. Killing tumour cells through the induction of apoptosis has been recognized as a novel strategy for the identification of antitumour drugs and a valuable tool for cancer treatment [9,10]. In this study, in order to determine the effect of the phenolic hydroxyl groups at C-4′ and C-7 of apigenin on its anticancer activity, 4′,7-diacetoxy apigenin [4-(7-acetoxy-5-hydroxy-4-oxo-4H-chromen-2-yl) phenyl acetate] was synthesized for the first time and its apoptotic induction in human Hep G2 cells was evaluated.

## 2. Results and Discussion

### 2.1. Identification of 4′,7-Diacetoxyapigenin

**Figure 1.**
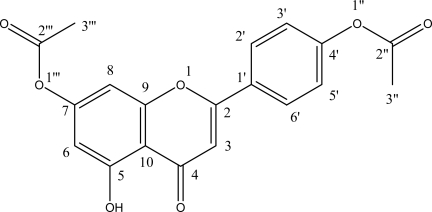
Chemical structure of 4′,7-diacetoxyapigenin.

Using UV, ESI-MS and NMR, the flavonoid obtained in this study were identified as 4′,7-diacetoxyapigenin (Figure 1).

### 2.2. Anti-Proliferation Activity of 4′,7-Diacetoxyapigenin on Hep G2 Cells

**Figure 2.**
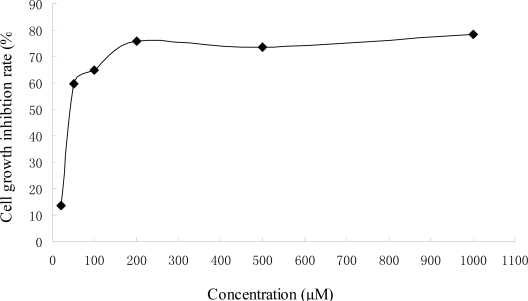
Effect of 4′,7-diacetoxyapigenin on Hep G2 cell proliferation.

**Figure 3.**
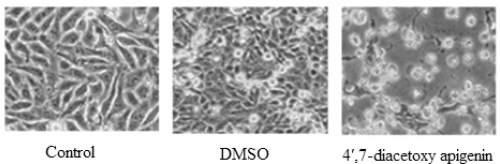
Morphology of Hep G2 cells treated by 4′,7-diacetoxyapigenin (70 μM, 48 h).

The inhibitory effects of 4′,7-diacetoxyapigenin on the proliferation of Hep G2 cells were tested at different concentration for 48 h and the inhibition rate (IR) was determined (Figure 2). It induced a dose-dependent inhibitory effect. The inhibitory concentration 50% (IC_50_) was about 73.7 μM. The effect of 4′,7-diacetoxy apigenin was far low than that of apigenin (about 29.7 μM) [11]. Apigenin was more potent in the inhibition of Hep G2 cells proliferation than that of 4′,7-diacetoxyapigenin, which could mean the presence of the phenolic hydroxyl groups at C-4′ and C-7 of apigenin was very important for its anticancer activiy. The effect of 4′,7-diacetoxyapigenin on the cell morphology is shown in Figure 3. It could be found that the viable cells could remain their common shape, but non-viable cells had lost their shape. And necrosis could also contribute to the induced cell death.

### 2.3. Flow Cytometry Analysis of Cell Cycle and Cell Apoptosis

To determine whether the Hep G2 cells treated with 4′,7-diacetoxyapigenin undergo apoptosis, the cell distribution in the cell cycle was examined by PI staining. The significant increase of the population in the G0/G1 phase in Hep G2 cells could be observed and the population of cells in other phases of the cell cycle remained unaffected.

**Figure 5.**
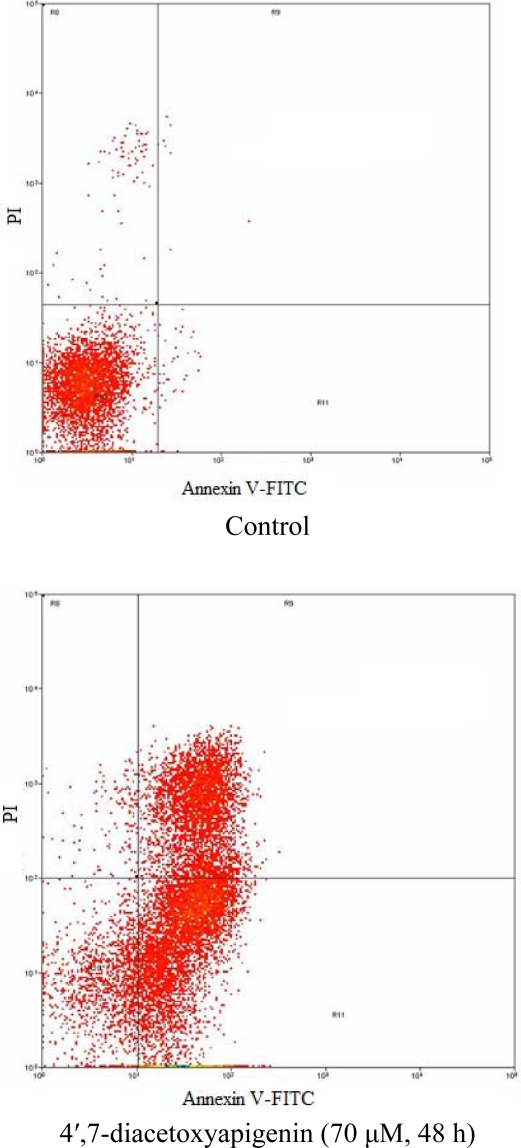
Flow cytometric analysis of phosphatidylserine externalization (annexin V binding) and cell membrane integrity (PI staining) in Hep G2 cells undergoing apoptosis.

The hallmark of early apoptotic cells is the transverse redistribution of plasma membrane phosphatidylserine (PS) [12]; thus, the annexin V binding assay was performed to detect the surface exposure of PS. In this study, the Hep G2 cells were treated with 4′,7-diacetoxyapigenin (70 μM) for 48 h. Figure 5 shows the FACS histogram with dual parameters including annexin V-FITC and PI. The dual parametric dot plots combining annexin V-FITC and PI fluorescence show the viable cell population in the lower left quadrant (annexin V-negative/PI-negative), the early apoptotic cells in the lower right quadrant (annexin V-positive/PI-negative), and the late apoptotic cells in the upper right quadrant (annexin V-positive/PI-positive). In untreated Hep G2 cells, 0.7% of cells were annexin V-positive/PI-negative, 0.1% of cells were both annexin V- and PI-positive. In treated Hep G2 cells, the annexin V-positive/PI-negative and double-positive cells increased to 54.1% and 32.9%, respectively. The result suggested that 4′,7-diacetoxyapigenin could induce cells into an apoptotic pathway resulting in the inhibition of proliferation of Hep G2.

## 3. Experimental Section

### 3.1. Materials and Instrumentation

Apigenin (Purity 95%) came from our previous study [13]. A human hepatoma cell line (Hep G2) was obtained from the Institute of Medicinal Biotechnology, Chinese Academy of Medical Sciences. Powdered Dulbecco modified eagle medium was purchased from GIBCO (Grand Island, NY, USA). Foetal bovine serum (FBS) and antibiotics (penicillin and streptomycin mixture) were purchased from Hyclone Laboratories, Inc. Propidium iodide (PI) and 3-[4,5-dimethylthiazol-2-yl]-2,5-diphenyl tetrazolium bromide (MTT) were purchased from Sigma Chemical Co. An annexin V-FITC apoptosis detection kit was purchased from CLONTECH Company. UV analysis was performed on a TU-1810PC UV-visible spectrophotometer (Purkinje, China). ^1^H- and ^13^C-NMR spectra were recorded in DMSO-d_6_ using an AVANCE Digital 400 MHz NMR spectrometer (Bruker, Germany). ESI-MS analysis was taken on a ZQ 2000 electrospray ionization mass spectrometer (Waters, USA) in the positive ion mode.

### 3.2. Synthesis of 4′,7-Diacetoxyapigenin

Acetic anhydride (0.94 mL, 10.0 mmol) was added dropwise to a solution of apigenin (1.35 g, 5.00 mmol) in dry pyridine (80 mL) at room temperature. After stirring the mixture for 24 h at room temperature, it is poured into ice-cold water (1,600 mL). The white precipitate is separated by filtration, washed twice with a small amount of ice-cold water and recrystallized from methanol (300 mL). The resultant material is washed with distilled water and dried at 60 °C to yield the product (0.842 g) as a slightly white solid. UV, λ_max_(nm) (MeOH) 270, 300sh; ESI-MS^2^ negative ion *m/z*: 747 ([2M+K]^+^), 731 ([2M+Na]^+^), 709 ([2M+H]^+^), 393 ([M+K]^+^), 377 ([M+Na]^+^), 355 ([M+H]^+^), 313 ([M-acetyl+H]^+^), 271 ([M-2acetyl+H]^+^); ^13^C-NMR δ, 183.02 (C-4), 169.35 (C-2″/2″′), 168.89 (C-2), 163.96 (C-5), 161.20 (C-7), 156.72 (C-9), 153.98 (C-4′), 128.65 (C-1′), 128.37 (C-2′/6′), 123.17 (C-3′/5′), 108.64 (C-6), 106.17 (C-10), 105.87(C-3),102.14 (C-8), 21.37 (C-3″/3″′); ^1^H-NMR δ, 12.87 (s, 5-OH), 8.16 (d, *J* = 8.79 HZ, H-2′/6′), 7.37 (d, *J* = 8.79 HZ, H-3′/5′), 7.14 (s, H-8), 7.10 (d, *J* = 1.99 HZ, H-3), 6.67 (d, *J* = 1.99 HZ, H-6), 2.32 (s, H-3″/3″′).

### 3.3. Cell Culture and Drug Treatment

Hep G2 cells were cultured in DMEM medium with 10% FBS, 100 UI/mL penicillin and 100 μg/mL streptomycin in humidified air at 37 °C with 5% CO_2_. Exponentially growing Hep G2 cells were collected and re-suspended in fresh medium for 4 h and then exposed to various concentrations of 4′,7-diacetoxy apigenin.

### 3.4. MTT Assay

Survival of cells was evaluated by using a system based on MTT, which was reduced by living cells to yield a soluble formazan product that could be detected colorimetrically. Cells were suspended in 96-well plates of 90 μL medium at a density of 2 × 10^4^ cells/well and 10 μL 4′,7-diacetoxyapigenin in different concentrations. These were then incubated in humidified air at 37 °C with 5% CO_2_ for 48 h, exposed to 10 μL MTT (5 mg/mL) and incubated for another 4 h under the conditions mentioned above. The formazan precipitate was dissolved in 100 μL DMSO. IC_50_ values were tested through the MTT method [14]. The inhibition rate (IR%) was calculated as follows: IR% = (mean control absorbance-mean experimental absorbance)/mean control absorbance ×100%.

### 3.5. Flow Cytometry Analysis

The flow cyctometry analysis was performed on a FACS Calibur Flow cytometer (BeckmanCoulter, USA). Cell pellets were fixed in 70% ethanol at −20 °C for at least 12 h or overnight. After being washed twice with ice-cold PBS, they were incubated in RNase A/PBS (1 mg/mL) at 37 °C for 30 min, and stained with PI (0.5 mg/mL) at room temperature for 15 min. The intracellular DNA was then labelled with PI and the PI fluorescence of individual nuclei determined by a FACSCalibur fluorescence-activated cell sorter at 488 nm excitation. Surface exposure of phosphatidylserine in apoptotic cells was measured by the annexin V-FITC apoptosis detection kit according to the manufacturer’s instructions. Additional exposure to PI made it possible to differentiate the early apoptotic cells (annexin V-positive/PI-negative) from the late apoptotic cells (annexin V-positive and PI-positive).

## 4. Conclusions

In this study, 4′,7-diacetoxyapigenin [4-(7-acetoxy-5-hydroxy-4-oxo-4H-chromen-2-yl) phenyl acetate] was synthesized for the first time. It could inhibit the proliferation of Hep G2 cells in a dose-dependent manner. By using flow cytometry, it was found that 4′,7-diacetoxyapigenin not only induced cells to enter into apoptosis, but also affected the progress of the cell cycle.

## Figures and Tables

**Figure 4.**
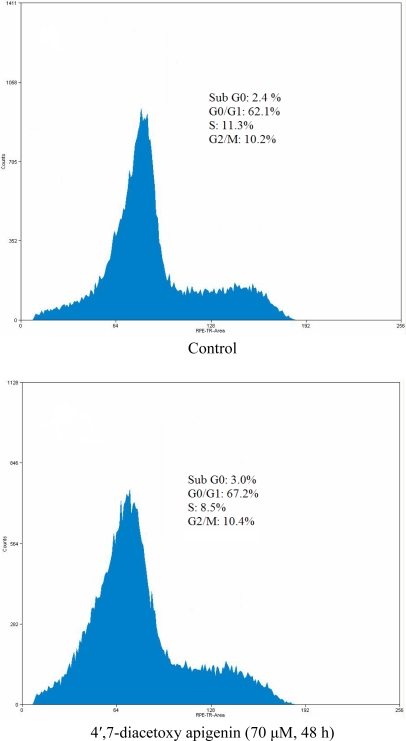
Effect of 4′,7-diacetoxyapigenin on cell cycle distribution in Hep G2 cells.
